# Implementation and One-Year Evaluation of Proenkephalin A in Critical Care

**DOI:** 10.3390/ijms26062602

**Published:** 2025-03-13

**Authors:** Lukas Martin, Caren Martin, Arne Peine, Matthias Imöhl, Alexander Kersten, Rafael Kramann, Turgay Saritas, Nikolaus Marx, Michael Dreher, Gernot Marx, Tim-Philipp Simon

**Affiliations:** 1 Department of Intensive Care Medicine, Faculty of Medicine, RWTH Aachen University, 52074 Aachen, Germany; 2 Laboratory Diagnostic Center, University Hospital RWTH Aachen, 52074 Aachen, Germany; 3 Department of Pneumology and Intensive Care Medicine, University Hospital RWTH Aachen, 52074 Aachen, Germany; 4 Department of Cardiology, Angiology and Internal Intensive Care Medicine, University Hospital RWTH Aachen, 52074 Aachen, Germany; 5 Department of Nephrology, Rheumatology, Clinical Immunology and Hypertension, University Hospital RWTH Aachen, 52074 Aachen, Germany

**Keywords:** proenkephalin A, proenkephalin A 119–159, PENK, kidney function biomarker, AKI, critical care

## Abstract

Proenkephalin A 119–159 (PENK) is a promising functional kidney biomarker, evaluated in various clinical settings. In critical care medicine, early diagnosis of acute kidney injury (AKI) is crucial; however, to date, the diagnosis and the assessment of kidney function is still based on serum creatinine (sCr) and urine output, both associated with several limitations. Between November 2020 and March 2022, we implemented PENK in our daily practice on our intensive care units (ICU). PENK, sCr, AKI stage, and the start and duration of renal replacement therapy (RRT) were documented. Almost 18,000 PENK measurements from 4169 patients were analyzed, and the glomerular filtration rate (GFR) was estimated with the new PENK-GFR formula. PENK outperformed sCR in the kidney function assessment and sCR trajectory over time. Moreover, PENK predicted the use of RRT and thus showed its usefulness in critical care daily practice.

## 1. Introduction

Acute kidney injury (AKI) affects about 15% of all hospitalized patients and up to 50–60% of intensive care patients. Additionally, it is associated with adverse long-term outcomes, including recurrent AKI episodes, re-admissions, cardiovascular events, and long-term mortality [1]. Furthermore, it can progress into chronic kidney disease (CKD)—with a pooled rate of 10.17 to 25.8 cases per 100 person-years [2,3]. A recent retrospective analysis from the Charité University Hospital (Berlin, Germany) found that all AKI stages were significantly under-coded and higher AKI stages were associated with increased length of stay, morbidity, and mortality [4]. Among ICU patients suffering from acute renal failure, the in-hospital mortality rate was 37%, and the rate of mortality or nonrecovery of renal function was 50% [5]. Additionally, knowing true kidney function is essential for pharmacotherapy, not only among ICU patients but also in other patients, as nephrotoxicity still accounts for around a fourth of serious side-effects after drug administration [6]. Therefore, early and validated detection of kidney function deterioration is crucial for initiating timely therapy and preventing potential chronic kidney damage or disease.

Nevertheless, today, the assessment of kidney function in the clinical setting and the definition of acute kidney injury (AKI) is based on serum creatinine (sCr) and urine output, for which clinicians and consensus groups agree that it is an imperfect gold standard. sCr rises relatively late after the onset of AKI and up to 50% of the nephrons might already be irreversibly damaged before an increase in sCr can be observed. Furthermore, sCr is influenced by multiple factors, including non-renal ones, such as gender, age, volume status, drugs, and muscle mass [7]. On the other hand, urine output, as the other most commonly used diagnostic parameter, can be misleading. Oliguria can occur as a physiologically adequate response that does not indicate a kidney dysfunction, while normal urine output can be preserved despite kidney damage—and therefore it lacks sensitivity and specificity [7].

As sCr and urinary output can be misleading, and kidney function can deteriorate rapidly and unnoticed, there is an urgent unmet need for more reliable diagnostics for kidney function, especially in critically ill patients. These limitations of today’s standard of care with sCr and urinary output have brought wide attention to the development of novel biomarkers to overcome these limitations and improve diagnostic accuracy of kidney function.

Renal biomarkers are divided into stress, damage, and functional markers, addressing different diagnostic questions. While stress and, especially, damage markers indicate cell stress or loss resulting in structural kidney damage, functional markers reflect changes in the glomerular filtration rate (GFR) [8]. Therefore, functional markers may offer the advantage of detecting significant deterioration of the kidney before actual severe damage occurs. Recent consensus from the Acute Disease Quality Initiative (ADQI) [8] recommends the additional use of biomarkers in clinical practice from AKI risk assessment, prediction, and prevention to diagnosis and management: About 20 stress and damage markers have been evaluated, including neutrophil gelatinase-associated lipocalin (NGAL), tissue inhibitor metalloproteinase-2 (TIMP-2)/insulin-like growth factor-binding protein 7 (IGFBP-7), and kidney injury molecule-1 (KIM-1). They may be used for specific indications to improve kidney diagnostics [9,10,11]. However, only proenkephalin A (PENK) 119–159 has been proposed as a novel functional biomarker—alongside sCr and Cystatin C (Cys C) [8]. While Cystatin C has found its way to clinical practice, PENK has not yet been established into clinical routine. This study aims to fill that gap.

PENK is a stable prohormone fragment of the unstable enkephalin, purely filtrated in the glomerulus and not secreted or absorbed in the tubules. It has been introduced as a precise and accurate renal functional biomarker. It has been evaluated in various clinical settings such as sepsis, acute heart failure, major surgery, severe burns, critical illness, renal transplantation, and liberation from renal replacement therapy (RRT), as well as in pediatric populations [12,13,14,15,16,17]. PENK values correlate with the severity of renal dysfunction, the onset of AKI, the need for RRT, and major acute kidney events (MAKE), including mortality [18]. While showing a clear kidney profile, there are some conditions that showed an influence on PENK level besides the kidney function, such as traumatic brain injuries, and ischemic or hemorrhagic strokes [19,20]. Moreover, decreased PENK has been reported in breast cancer patients and women at risk of developing breast cancer [21,22]. Commercial tests for the quantitative determination of PENK are available for point-of-care use and as manual chemiluminescence-based immunoassays.

In various populations, PENK concentrations have shown a stronger correlation with the measured glomerular filtration rate (mGFR) than sCr-based methods [18]. This laid the groundwork for the development of a formula for the estimation of GFR (eGFR) based on PENK to overcome known limitations [16,17,18,23]. A recently published study incorporating PENK values into the calculation of eGFR has shown superiority over commonly applied formulas including MDRD (Modification of Diet in Renal Disease) and CKD-EPI (Chronic Kidney Disease Epidemiology Collaboration), especially in the range of estimating GFR between 30 and 90 mL/min/1.73 m^2^ [24].

In the past decade, over 4500 publications addressing the topic of “novel kidney biomarkers” have been released. However, despite being extensively studied, novel biomarkers are rarely implemented into clinical practice. Some of the reasons may be the lack of data and validation in relevant indications or on validated platforms. However, it is likely that multiple obstacles downstream of the verification process prevent innovations, in general, from actually being used in daily practice [25].

Based on the solid scientific evidence for PENK as a timely biomarker for GFR estimation, we therefore decided to implement and investigate this parameter in our daily practice in the ICU. Supported by data from over 4000 patients, this work provides—for the first time—evidence for the use of PENK in day-to-day practice and paves the way for further evaluation to confirm the existing scientific evidence for PENK in a routine setting.

## 2. Results

### 2.1. Implementation

After a preparation phase, including regular trainings of attending physicians, residents, and nursing staff, we started measuring PENK in our diagnostic morning routine. The technology—based on a manual assay format [23]—was established in the central laboratory, which enables a high sample throughput in an adequate time. Although the measurement lacks full automation, an optimization of the process, including automatic sorting of EDTA plasma samples via automated hematology systems and automated pipetting, makes the daily use feasible and reduces hands-on-time to 3 h. PENK results were reported before the morning ward round and available for the decision on daily treatment protocols.

### 2.2. Baseline Characteristics

During the study period, over 17,000 PENK measurements were performed by the central laboratory. A total of 4169 patients (64% males, median age 66 years, median body mass index 26.4 kg/m^2^) had at least one available PENK value and were included into the analysis. Demographic and clinical characteristics of these patients are listed in Table 1. Based on the rise of sCr as part of the KDIGO criteria, the proportion of patients with AKI stage 1 was 5.3% and 6.8% for stage 2 or 3, while 9.9% received RRT (in accumulation for a total of 3712 days) while in the ICU. Of the n = 4169 patients included in the analysis, 35% stayed up to 2 days in the ICU, 35% between 3 and 7 days, and the remaining patients stayed longer or were re-admitted during the observation period.

### 2.3. Correlation of PENK, sCr, and eGFR

PENK and creatinine in all measured samples correlated moderately well (Spearman r = 0.54, n = 17,900, *p <* 0.001, pairwise complete samples only), and so did the different eGFR formulas investigated (Table 2).

### 2.4. Initial Risk Stratification at ICU Admission

**Figure 1:**
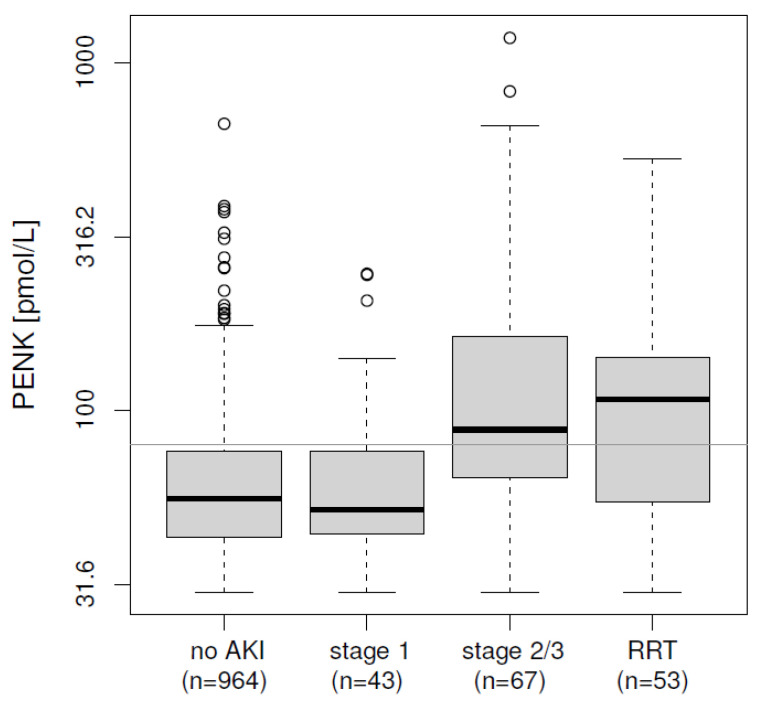
PENK concentration on admission to ICU, grouped by maximum AKI stage reached or need of RRT during ICU stay (*p <* 0.0001); horizontal line at 80 pmol/L. Initial PENK levels increased with the severity of AKI stage that patients reached during their stay in the ICU. AKI, acute kidney injury; PENK, proenkephalin A 119–159; RRT, renal replacement therapy.

PENK and sCr from the day of ICU admission were available in n = 1058 patients, of which n = 101 developed an AKI (KDIGO stage 2 or 3) or required RRT during the ICU stay. In these samples, PENK levels increased with the severity of AKI (*p <* 0.0001, Figure 1). PENK varied from a median (IQR) of 56 (43–76) pmol/L for patients without kidney dysfunction and 52 (44–76) pmol/L in KDIGO stage 1 to 88 (64–163) pmol/L for patients with KDIGO stage 2 or 3, and 108 (55–142) pmol/L for patients requiring RRT. In all patients, both PENK and sCr at admission predicted KDIGO stage 2/3 or requirement of acute RRT equally well (AUC 0.725 and 0.716 for PENK and sCr, respectively, both *p <* 0.0001, see Table 3). Additionally, both markers provided added value on top of each other (added value via nested regression models, see methods for details, *p =* 0.0001).

### 2.5. Biomarker Dynamics: Predicting AKI in Cases with Low sCr (<1.2 mg/dL) on Admission

To evaluate our hypothesis that PENK is earlier and more dynamic than sCr, we investigated several approaches. First, we compared the trajectories of both markers in patients with normal sCr (<1.2 mg/dL) at ICU admission. Of these patients (n = 910), 56 developed AKI stage 2/3 or required acute RRT during ICU stay. PENK outperformed sCr in prediction (AUC 0.650, *p =* 0.0002 vs. AUC 0.530, *p =* 0.6556, respectively; *p =* 0.0002 for added value of PENK). This was also true when measured at 24 h and 48 h post-ICU admission (both *p <* 0.0001, Table 3).

**Table 3:** Initial risk stratification for AKI: prediction of KDIGO stage 2/3 or need for RRT. PENK, sCr and estimated GFRs of all patients at timepoint admission and of patients (with a low sCr < 1.2 mg/dL at admission) at the three timepoints: admission, 24 h, and 48 h after admission. All AUC > 0.5 with *p <* 0.05 except for sCr and CKD-EPI in patients with sCr < 1.2 mg/dL on admission. PENK is superior to sCr in patients with sCr < 1.2 mg/dL on admission at all three timepoints and provides added value on top of sCr in all patients (all *p <* 0.001). With an AUC larger than 0.8 at 24 h and 48 h (PENK and eGFR versions including PENK), the biomarker allows identification of AKI in patients with low sCr upon admission. AKI, acute kidney injury; AUC, area under the receiver operating curve; CI, confidence interval; sCr, serum creatinine; eGFR, estimated glomerular filtration rate; KDIGO, Kidney Disease: Improving Global Outcomes; PENK, proenkephalin A 119–159; Crea, creatinine.

	Admission, All Patients		Admission, sCr_adm_ < 1.2 mg/dL		24 h, sCr_adm_ < 1.2 mg/dL		48 h, sCr_adm_ < 1.2 mg/dL	
	n (Events)	AUC [95% CI]	n (Events)	AUC [95% CI]	n (Events)	AUC [95% CI]	n (Events)	AUC [95% CI]
PENK	1127 (120)	0.725 [0.67, 0.78]	910 (56)	0.65 [0.565, 0.735]	326 (27)	0.818 [0.719, 0.918]	175 (22)	0.877 [0.795, 0.959]
sCr	1127 (120)	0.716 [0.659, 0.772]	910 (56)	0.53 [0.447, 0.615]	326 (27)	0.689 [0.575, 0.803]	175 (22)	0.74 [0.625, 0.856]
eGFR_CKD-EPI_	1058 (101)	0.751 [0.697, 0.806]	857 (45)	0.603 [0.52, 0.687]	293 (20)	0.769 [0.647, 0.891]	154 (15)	0.796 [0.652, 0.94]
eGFR_PENK-Crea_	1058 (101)	0.755 [0.699, 0.811]	857 (45)	0.624 [0.529, 0.719]	293 (20)	0.83 [0.724, 0.937]	154 (15)	0.908 [0.842, 0.974]
eGFR_PENK_	1058 (101)	0.728 [0.668, 0.788]	857 (45)	0.638 [0.542, 0.734]	293 (20)	0.807 [0.684, 0.929]	154 (15)	0.862 [0.772, 0.952]

**Figure 2:**
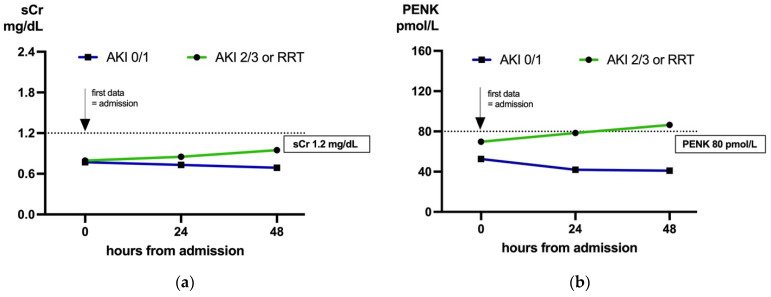
Patients with sCr < 1.2 mg/dL on admission, stratification by maximum AKI stage during ICU stay: KDIGO stage 0/1 (blue) vs. KDIGO stage 2/3 or need for RRT (green). (**a**) sCr levels at 0, 24, and 48 h from admission. (**b**) PENK levels at 0, 24, and 48 h from admission. Median sCr and PENK plotted. PENK is superior to sCr at all three timepoints (*p*-value for added value < 0.001, while *p*-value for sCr >0.05 at all three timepoints). See Table 3 for sample size per group and time point. AKI, acute kidney injury; sCr, serum creatinine; ICU, intensive care unit; KDIGO, Kidney Disease: Improving Global Outcomes; PENK, proenkephalin A 119–159; RRT, renal replacement therapy; Crea, creatinine.

To illustrate the different trajectories, we plotted the median biomarker concentration in patients with sCr < 1.2 mg/dL from admission, as well as 24 h and 48 h post admission, stratified by KDIGO stage 0/1 vs. KDIGO stage 2/3 or the need for RRT (Figure 2).

Likewise, both newly proposed eGFR formulas including PENK by [24] outperformed the latest CKD-EPI version [26] in patients with low sCr on admission at all three time points (all *p <* 0.05, Table 3). In fact, when estimated with the CKD-EPI formula, no significant difference of median eGFR values was observed on the day of admission (*p =* 0.062), but in patients with KDIGO stage 2/3 or the need for RRT, the median eGFR values declined in the following days. In comparison, median eGFR based on PENK and age (eGFR_PENK_) differed significantly on admission (*p =* 0.004) and clearly separated patients at 24 h and 48 h (Figure 2).

### 2.6. Biomarker Dynamics: Trajectories Post-sCr Peak

Our second approach evaluated whether PENK concentrations precede the rise or decline in sCr. For this approach, we defined time point 0 as the first time of observing an sCr value > 1.2 mg/mL within the first 14 days after initial ICU admission (with this time limit chosen to exclude data from re-admission and long-term ICU patients). Of all patients admitted to the ICU, n = 367 met the criteria, and corresponding PENK and sCr values were available. In retrospect, 30% of these patients had already exceeded the clinical cut-off for PENK of 80 pmol/L at least 24 h earlier, 16% had exceeded it 48 h earlier, and 7% had exceeded it 72 h earlier.

**Figure 3:**
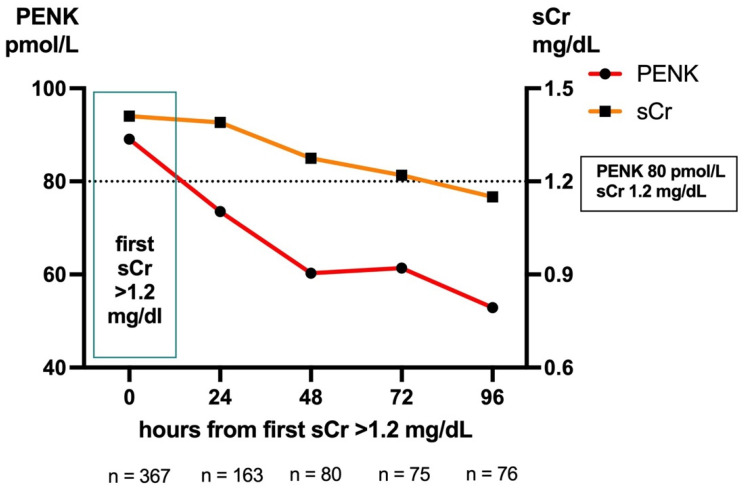
Biomarker trajectories of PENK and sCr measured after first sCr rise above 1.2 mg/dL. with the timepoints at 0, 24, 48, 72, and 96 h. Included patients did not receive RRT at a later time point. sCr, serum creatinine; PENK, proenkephalin A 119–159.

To examine trajectories after the initial rise in sCr > 1.2 mg/mL, patients who received RRT after this time point were excluded to avoid biasing sCr values (which would falsely decline due to RRT). In the included patients, median PENK values fell below the clinical cut-off of 80 pmol/L within one day, while median sCr values did not fall below the clinical cut-off of 1.2 mg/dL until day four (Figure 3). Although the difference in trajectories between PENK and sCr within 24 h, 48 h, 72 h, and 96 h did not reach statistical significance (all *p* > 0.05), the observed trend illustrates that PENK provides critical information earlier than sCr.

### 2.7. Kidney Function During RRT

**Figure 4:**
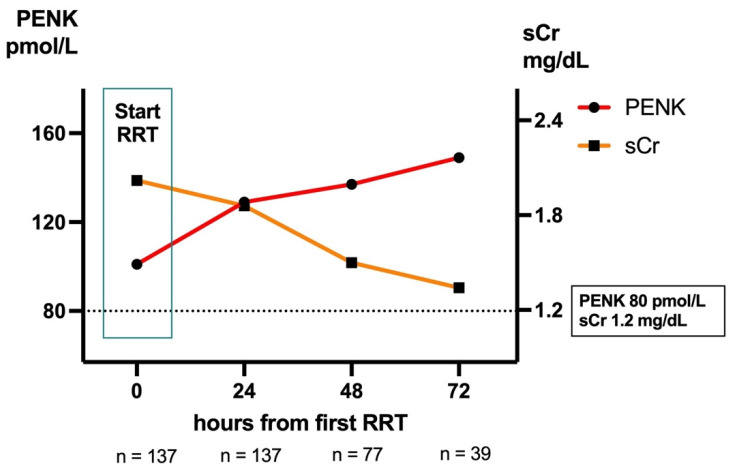
Biomarker trajectories in patients receiving RRT. Only patients included with both PENK and creatinine measurements available at start of RRT (0 h) and 24 h (n = 137). Change in PENK within 24 h (n = 137), 48 h (n = 77), and 72 h (n = 39) differed substantially from change in creatinine (all *p <* 0.0001). sCr, serum creatinine; PENK, proenkephalin A 119–159; RRT, renal replacement therapy.

To compare biomarker trajectories after initiating RRT, we compared PENK and sCr in patients receiving RRT from the day of the first RRT to 72 h post-RRT initiation (irrespective of the duration of RRT). Only patients with valid biomarker data from the day of RRT initiation and 24 h later were included (n = 137) to evaluate initial changes in both biomarkers. Changes in PENK within 24 h, 48 h, and 72 h differed significantly from changes in sCr (all *p <* 0.0001; Figure 4). While both peptides were filtered by RRT [27], only sCr declined during RRT: from 2.02 mg/dL (IQR 1.52–2.89) on the day of RRT initiation to 1.86 mg/dL (1.23–2.50) 24 h later, 1.50 mg/dL (0.95–2.2) 48 h later, and 1.34 mg/dL (0.93–2.10) 72 h later. PENK, on the other hand, continued to increase in these patients: from 102 pmol/L (63–173) on the day of RRT initiation to 129 pmol/L (83–184) 24 h later, 137 pmol/L (83–203) 48 h later, and 149 pmol/L (97–185) 72 h later. Hence, while falling sCr values would have suggested kidney function improvement, PENK suggested otherwise.

### 2.8. Comparison of eGFR During RRT by Net Reclassification Index (NRI)-like Methods

To confirm superior performance of PENK over sCr during RRT, another statistical method was applied based on the inclusion of all patient samples and the application of NRI-like methods to evaluate correctness. As this also requires simplification of the measured data, this approach is rougher than the previous one. We applied it to the GFR version based on CKD-EPI and the formula based on PENK and age (eGFR_PENK_) for illustration. Both eGFRs could be estimated in n = 15,819 patient samples. To determine which of the two GFR estimates better reflects the true GFR, we divided the patient samples into three different groups: 12,120 samples were from patients who never received RRT, where higher GFR estimates would be assumed to be more correct; 1945 samples were either from pre- or post-RRT days from RRT patients, where lower GFR estimates would be assumed to be correct; and 1819 samples were taken on days with RRT, where—again—a lower GFR would be assumed to be correct. This approach allowed an evaluation of the two methods similar to the underlying concept of the NRI [28]. Table 4 compares eGFR_CKD-EPI_ and eGFR_PENK_ below/above 60 mL/min/1.73 m^2^ for all patients, as well as separately for each of the three groups defined. Consistent with our assumption that higher GFR estimates are more accurate in patients without the need for RRT, the PENK formula was more accurate in 0.7% of these samples. For patients in need of RRT and samples collected the day before or after RRT, the PENK formula was more accurate in 1.7% of these samples. Finally, in samples taken on RRT days, where sCr is expected to give incorrect estimates due to filtering, the PENK formula was more accurate in 15.1% of these samples.

## 3. Discussion

Assessing kidney function is essential, especially in ICU patients who may develop an acute kidney injury (AKI) in up to 50–60% of the cases [1], and a mortality of up to 46% in those requiring RRT [29]. Additionally, accurately assessing kidney function is essential for treating the underlying causes of AKI, such as sepsis [30], trauma [31], post-cardiac surgery [32], or acute coronary syndrome [33]—to name only a few. Early detection of deterioration is crucial for knowing the exact kidney function, and new biomarkers have been studied for this purpose. Finally, every pharmacotherapy has the risk of nephrotoxicity, which requires a close monitoring of the kidney function [6]. Nevertheless, the kidney function assessment and diagnosis of an AKI in clinical routine is still based on sCr and urine output nowadays, which lack sensitivity and specificity [7]. We were motivated by Ostermann et al., who, in 2020, recommended testing alternatives to sCr in clinical practice and encouraged the adoption of novel biomarkers that had been extensively studied in clinical trials but were still not implemented to change our diagnostic view on kidney function [8]. However, the implementation of new diagnostics is crucial to translate innovation into practice, generating clinical familiarity and phenotypes.

Among more than 20 new biomarkers which were evaluated by the ACQI, only sCr (with its known limitations), Cystatin C, and PENK have been recommended as functional kidney markers [8]. Cystatin C has found widespread application in chronic kidney disease settings. While our nephrology department has been using Cystatin C in addition to sCr, it has not been implemented in our ICU due to its limitations in critically ill patients, such as being influenced by age, gender, race, diabetes, steroid treatments, thyroid dysfunction, and inflammation [34,35,36,37].

Therefore, we chose PENK as an alternative renal function marker as studies have proven its validity in critical care patients [38] including patients with sepsis [13], acute heart failure [14], major surgery [39], and severe burns [40]. All studies have shown an independent association with renal endpoints, including sub-AKI, AKI in 48 h and 7 days, requirement of RRT, and major acute kidney events (MAKE), including mortality. PENK levels have been shown to precede changes in sCr up to 48 h [13] and have been shown to correlate well with the measured GFR assessed by iohexol or iothalamate methods both in stable and unstable patients [41]. This also applies to neonates and children, where the correlation of PENK with measured GFR has been shown to outperform that of sCr and Cystatin C [17]. Those results were a prerequisite for the application of biomarker-based GFR calculations, which have been developed by Beunders et al. and have shown superiority over the existing estimations based on sCr, namely CKD-EPI and MDRD [24].

Our results support the findings of previous studies and the viability of PENK as a biomarker to be applied in intensive care patients in daily practice. PENK overcomes the limitations of sCr, namely the delay in concentration changes, its influence by non-renal factors, and its limitations in patients undergoing RRT.

Our data prove that PENK allows a significantly better assessment of renal dysfunction on admission and better identifies patients who will develop severe AKI KDIGO stage 2 or 3 or that require RRT during the ICU stay. Especially in patients with a normal sCr (<1.2 mg/dL) at admission, whose renal function might be overestimated, PENK and the new eGFR formula based on PENK outperformed sCr and the eGFR_CKD-EPI_ in the prediction of an AKI (stage 2/3 or RRT requirement) on admission, at 24 h, and at 48 h later, respectively. This way, PENK may overcome the critical time gap of sCr.

An earlier knowledge of a patient’s compromised renal function allows a timely intervention, preventing progression to more severe stages, which reduces the risk for emergency RRT and improves the chance for full renal recovery [42]. Such interventions may include the consultation of a nephrologist and application of the KDIGO bundles [43], thereby supporting appropriate resource allocation, proper coding of patients, and potentially reducing the length of the ICU stay.

The slow dynamics of sCr have been a major criticism of the biomarker in critical care diagnostics. Therefore, several concepts of sub-AKI have been published in an effort to address the issues of AKI definition in clinical practice, as well as an adequate endpoint in clinical studies [44]. One may question if novel biomarkers, including those of renal damage [9] and renal function [12,45], predict AKI or rather display the actual renal impairment at the time of determination, knowing that sCr only rises when about 50% of all kidney function is already compromised [46]. Thus, especially in critical care when patient status is changing rapidly, the time delay of sCr carries the risk of overestimating renal function. Vice versa, recovering renal function may only be detected with a substantial time delay. Comparing the dynamics of sCr and PENK in our data shows that PENK precedes sCr changes. Fast dynamics of organ-specific biomarkers and an early inclusion of renal function as a factor into risk–benefit decision-making help improve treatment protocols, e.g., with respect to the subscription of known nephrotoxic drugs, such as chemotherapeutics for multimodal cancer treatment as well as commonly used antibiotics (e.g., aminoglycosides) and diuretics [8]. Earlier knowledge of renal recovery may also allow for the de-escalation of treatments, prevention of unnecessary RRT, and reduction in the length of ICU stays.

Whilst several large trials have investigated the optimal timing to initiate RRT in critically ill patients [47,48], studies to investigate parameters that can guide discontinuation from RRT are still lacking [49]. Currently, only a recommendation to discontinue RRT when no longer required—either because intrinsic kidney function has recovered to the point that it is inadequate to meet patient needs, or because RRT is no longer consistent with the goals of care—is available [43]. As creatinine and urea are filtered and influenced by RRT, the most used predictor of recovering renal function in patients undergoing RRT is urine output, which remains a subject of controversy [50,51]. In practice, this lack of objective parameters to guide RRT discontinuation may cause unsuccessful weaning and the repeated need to initiate RRT as renal function has not yet recovered. Each RRT cycle carries relevant risks for complications such as air embolism, electrolyte or fluid abnormalities, hypothermia, infections, or bleeding, or the so-called CRRT- or dialytrauma [16,52,53].

Our data suggest that PENK levels remain dynamic and increase during the first 72 h of RRT. Additionally, PENK levels fell faster compared to sCr in the first 96 h after the initial rise of sCr above 1,2 mg/dL in patients not requiring RRT, even though it did not reach significance. PENK should be effectively removed via RRT [27]. Hence, persistently elevated PENK during RRT may be an indication of ongoing renal dysfunction, while declining PENK levels may serve as an indicator for renal recovery, as suggested by the recent findings from the post hoc analysis of the single-center ELAIN trail [16], the multicenter RICH trial [54], and Tichy’s (2024) prospective study [55] on early and successful liberation from RRT in critically ill patients with AKI. The data imply that PENK cannot simply be a biomarker of renal filtration but may be directly or indirectly related to molecular mechanisms that stimulate kidney function via its related enkephalins, for example by inducing natriuresis and diuresis [56,57] even though the exact mechanisms remain unclear at present [58]. Assessing patients’ GFR by PENK while on RRT may hold the potential to improve patient management and reduce the risks of RRT, for example by limiting the treatment time to the minimum required, as suggested by [16].

## 4. Materials and Methods

### 4.1. Patient Population

All patients admitted to the intensive, weaning, or intermediate care units of the University Hospital RWTH Aachen, Germany, between November 2020 and March 2022, have been included in the study. The patient population was made up of perioperative, cardiology, and pneumology patients. Only patients without any PENK measurements (for example due to a short ICU stay on a weekend, when PENK measurements were not available) were excluded from the study.

### 4.2. Ethics Approval and Data Collection

The analysis was approved by the local ethics committee (EK122/13) and the study was conducted in accordance with Directive 2001/20/EC as well as the Good Clinical Practice Guidelines (ICH version 4 of 1 May 1996, and Decision of 24 November 2006) and the Declaration of Helsinki. Parameters including demographics (age, gender), sCr, PENK, as well as start and duration of RRT were extracted from the patient data management system (Intellispace Critical Care and Anesthesia (ICCA), Version J.01.00, Philips, Eindhoven, The Netherlands).

### 4.3. Implementation Process

We have implemented PENK in a routine setting by a carefully led and supervised change management process in the health care sector, orienting on Lewin’s model (unfreeze, move, freeze) [59,60]. Precisely, the integration of PENK into clinical practice was structured as follows:

1. Unfreeze: This stage involved preparing the institution for change by addressing the need for PENK for kidney function diagnostics. The head of the department played a key role in initiating discussions, securing approvals, and ensuring alignment with institutional goals. Attending physicians were engaged early to recognize the clinical value of PENK, and initial training sessions began to educate healthcare staff. Additionally, discussions with the laboratory, IT, and procurement teams took place to assess feasibility and prepare for technical implementation.
2. Move: During this phase, the actual implementation of PENK took place. Training and education were intensified to ensure that physicians accurately interpret and utilize PENK in clinical workflows. PENK was formally integrated into standard operating procedures (SOPs) to ensure consistent application. Simultaneously, technical implementation occurred, involving assay validation in collaboration with the laboratory, integration into electronic healthcare systems by the IT department, and procurement securing necessary reagents and equipment. PENK was then actively used in clinical settings, and preliminary data collection began to monitor its effectiveness.
3. Freeze: In the final stage we assessed PENK’s clinical value. Additionally, the experience in regards to feasibility from all involved departments was taken into account to evaluate and optimize the process.

### 4.4. Measurement of PENK and Other Variables

Blood samples were taken on weekdays as part of the morning routine and the PENK analysis was carried out in the central laboratory together with standard parameters. PENK was measured in EDTA plasma with a quantitative one-step chemiluminescence sandwich immunoassay (sphingotest^®^ penKid^®^, SphingoTec GmbH, Hennigsdorf, Germany). In brief, reagents and centrifuged monovettes were loaded into an automated pipetting robot Dynex DS2 (Dynex Technologies, Chantilly, VA, USA) and PENK measurements were performed in duplicates using 50 µL per well. Samples were automatically pipetted onto a microtiter plate coated with monoclonal antibodies against PENK and incubated for 1 h with 150 µL of detection antibody. Synthetic human PENK was used for calibration. After washing, the chemiluminescence signal was measured in a microtiter plate luminescence reader (Centro LB960, Berthold Technologies, Bad Wildbad, Germany). The lower detection limit of the assay was 29.9 pmol/L. In a reference population of 100 healthy subjects, the median (95th percentile) PENK level was 48.1 pmol/L (83 pmol/L) [23]. PENK results were directly documented in the local patient data management system ICCA (Philips, Eindhoven, The Netherlands). All other data analyzed were part of the routine data entries in the electronic patient records.

### 4.5. Statistics

Values are expressed as medians and interquartile ranges (IQR) or counts and percentages as appropriate. Group comparisons of continuous variables were performed using the Kruskal–Wallis test. Additional methods included receiver operating characteristic (ROC) curves to evaluate dichotomous endpoints (*p*-values based on logistic regression for greater sensitivity) and the comparison of correlated ROC curves via nested logistic regression models (i.e., comparing models including the individual markers with the model including both markers), as well as Spearman rank–order correlation. Biomarker data were log-transformed for statistical analysis, and non-parametric methods were applied due to the log-normal distributions of the biomarkers, unless otherwise specified.

In addition to the latest estimated GFR (eGFR) formula from the Chronic Kidney Disease Epidemiology Collaboration (eGFR_CKD-EPI_) [26], two new formulas for eGFR were applied to our data set. The first (eGFR_PENK-Crea_) was proposed by [24] and combines PENK, sCr and age. The second (eGFR_PENK_) excludes sCr and is intended to be used in situations where sCr is known to be unreliable, e.g., in patients under RRT [24]. The two new GFR formulas, using creatinine in µmol/l, age in years, and PENK in pmol/L, are as follows:

eGFR_PENK-Crea_ = 84 + 72.5 × tanh[ 5.5 − 1.10 × log10(PENK) − 1.29 × log10(sCr) − 0.63 × log10(Age)]

eGFR_PENK_ = 10^[ 3.79 − 0.777 × log10(PENK) − 0.324 × log10(Age)]

For initial risk stratification, we investigated the endpoints KDIGO stage 2/3 (Kidney Disease: Improving Global Outcomes) [43] or the need for RRT versus KDIGO stage 0/1 in patients with available PENK and sCr data from the day of ICU admission. To compare biomarker trajectories, we applied a paired *t*-test to test (as the normality assumption was not violated in this case; tested with the Shapiro-Wilk test) whether the relative change of sCr within 24 h, 48 h, 72 h, or 96 h from the respective starting point differed from the corresponding relative change of PENK. For illustration, we marked and, in some analyses, applied the following cut-off points: 80 pmol/L for PENK (approximately corresponding to the upper normal range reported at 83 pmol/L [23]), 1.2 mg/dL for sCr, and 60 mL/min/1.73 m^2^ for all eGFR versions.

## 5. Conclusions

This work underlies the well-known and urgent unmet need for innovative biomarkers that reflect kidney function, particularly in critically ill patients. It confirms—for the first time—the successful implementation and existing evidence on PENK in daily practice. Thus, our work not only paves the way for bringing innovation into clinical daily practice but at the same time fosters the translation of the evidence on PENK as a promising biomarker for kidney function into clinical routine.

Based on the solid scientific evidence for PENK as a timely biomarker for GFR estimation, we decided to implement and investigate this parameter in our daily practice in the ICU. Supported by data from over 4000 patients, we herein provide evidence for the usefulness of PENK in daily practice as well as its superiority over sCr in the timely assessment of kidney function, and in patients undergoing RRT.

Our real-world data provide a robust foundation for a comprehensive health economic study, which we plan to conduct as the next step to assess the clinical significance of PENK in relation to its testing costs and long-term economic impact.

We also strongly encourage the further evaluation of PENK in routine settings to expand the database and further confirm the scientific evidence, for example in multi-center studies.

## 6. Limitations

One of the strengths of our evaluation is the application in routine use, which is mandatory to better understand the application of novel biomarkers and required to gain clinical familiarity. However, as our report contains routine data, comparative analysis had to be limited to the number of patients and samples with data available in both sCr and PENK. Unavailability of PENK measurements during weekends additionally reduced the dataset included in this study. Bias may also occur as PENK results were part of clinical decision-making. Moreover, the study did not focus on the evaluation of potential confounders, e.g., patient variability due to the real world evidence setting without a specific study population. In addition, the complex medication regimes in intensive care settings, with their associated impact on factors such as volume distribution and organ function, might be an additional confounding factor. Comparing biomarker dynamics in clinical settings is challenging, especially if patients are not standardized. Hence, while our results indicate that PENK is earlier and more dynamic than creatinine, further research in more controlled settings is warranted to clinically prove this beyond doubt.

## Figures and Tables

**Table 1:** Demographics and clinical characteristics in median (IQR) or % of all included patients (n = 4169) admitted to ICU between November 2020 and March 2022, including maximum AKI stage reached during the ICU stay. AKI, acute kidney injury; BMI, body mass index; ICU, intensive care unit; IQR, interquartile range; KDIGO, Kidney Disease: Improving Global Outcomes; RRT, renal replacement therapy.

Characteristics (n = 4169 Patients Included)		Median (IQR)
General	Age, years	66 (56–75)
	BMI, kg/m^2^	26.4 (23.9–30.1)
		**in %**
Gender	Female	36%
	Male	64%
**Maximal AKI stadium during ICU stay**	No AKI	77.9%
	KDIGO 1	5.3%
	KDIGO 2 or 3	6.8%
	RRT	9.9%
Length of ICU stay	Less than 2 days	35%
	3–7 days	35%
	8–14 days	13%
	15–28 days	10%
	>28 days or re-admitted	7%

**Table 2:** Spearman correlation coefficient r of PENK, sCr, and eGFR versions investigated: All available PENK measurements from all consecutive patients included; all *p <* 0.001. n indicates the number of samples available with both measurements/estimates for the respective correlation analysis. CI, confidence interval; eGFR, estimated glomerular filtration rate; PENK, proenkephalin A 119–159; sCr, serum creatinine.

	PENK	sCr	eGFR_CKD-EPI_	eGFR_PENK-Crea_	eGFR_PENK_
PENK	-	r = 0.54 (CI 0.53, −0.55)	r = −0.62 (CI −0.63, −0.61)	r = −0.85 (CI −0.85, −0.84)	r = −0.98 (CI −0.98, −0.98)
sCr	n = 17,900	-	r = −0.91 (CI −0.91, −0.90)	r = −0.87 (CI −0.88, −0.87)	r = −0.55 (CI −0.56, −0.54)
eGFR_CKD-EPI_	n = 15,819	n = 15,819	-	r = 0.91 (CI 0.9, 0.91)	r = 0.68 (CI 0.67, 0.69)
eGFR_PENK-Crea_	n = 15,819	n = 15,819	n = 15,819	-	r = 0.87 (CI 0.87, 0.88)
eGFR_PENK_	n = 15,819	n = 15,819	n = 15,819	n = 15,819	-

**Table 4:** Comparison of GFR estimates based on CKD-EPI and on the new PENK formula. Analysis conducted for all patient samples (All) as well as for the three distinct subgroups of all samples from patients who did not require RRT (no RRT), of all samples taken before or after RRT (with RRT), and of all samples from days with RRT (RRT days). CKD-EPI, eGFR_CKD-EPI_; PENK, proenkephalin A 119–159; RRT, renal replacement therapy.

All n = 15,819	eGFR_PENK_		No RRT, n = 12,120	eGFR_PENK_		With RRT, n = 1945	eGFR_PENK_		RRT Days, n = 1754	eGFR_PENK_	
eGFR_CKD-EPI_	<60	>60	eGFR_CKD-EPI_	<60	>60	eGFR_CKD-EPI_	<60	>60	eGFR_CKD-EPI_	<60	>60
<60	3249	564	<60	1439	422	<60	702	91	<60	1108	51
>60	782	11,224	>60	343	9916	>60	124	1028	>60	315	280
	**n**	**%** **total**		**n**	**%** **total**		**n**	**%** **total**		**n**	**%** **total**
higher in eGFR_PENK_	564	3.6%	higher in eGFR_PENK_	422	3.5%	lower in eGFR_PENK_	124	6.4%	lower in eGFR_PENK_	315	18.0%
higher in eGFR_CKD-EPI_	782	4.9%	higher in eGFR_CKD-EPI_	343	2.8%	lower in eGFR_CKD-EPI_	91	4.7%	lower in eGFR_CKD-EPI_	51	2.9%
Delta	218	1.4%	Delta	79	0.7%	Delta	33	1.7%	Delta	264	15.1%

## Data Availability

All data is available upon request and in line with ethical and legal limitations.

## Abbreviations

The following abbreviations are used in this manuscript:

ADQI	Acute Disease Quality Initiative
AKI	Acute kidney injury
AUC	Area under the receiver operating curve
BMI	Body mass index
CI	Confidence interval
CKD	Chronic kidney disease
CKD-EPI	Chronic Kidney Disease Epidemiology Collaboration
Crea	Creatinine
CRRT	Continuous renal replacement therapy
Cys C	Cystatin C
EDTA	Ethylenediaminetetraacetic acid
eGFR	Estimated glomerular filtration rate
eGFR_CKD-EPI_	Estimated GFR based on *Chronic Kidney Disease Epidemiology Collaboration*
eGFR_PENK-Crea_	Estimated GFR based on Proenkephalin A 119–159, age and serum creatinine
eGFR_PENK_	Estimated GFR based on Proenkephalin A 119–159 and age
EK	Ethikkommittee
GFR	Glomerular filtration rate
ICCA	Intellispace Critical Care and Anesthesia
ICH	International Council for Harmonisation of Technical Requirements for Registration of Pharmaceuticals for Human Use
ICU	Intensive care unit(s)
IGFBP-7	insulin-like growth factor-binding protein 7
IQR	Interquartile ranges
KDIGO	Kidney Disease: Improving Global Outcomes
KIM	kidney injury molecule-1
MAKE	Major acute kidney events
MDRD	Modification of Diet in Renal Disease
mGFR	Measured glomerular filtration rate
NGAL	neutrophil gelatinase-associated lipocalin
NRI	Net Reclassification Index
PENK	Proenkephalin A 119–159
ROC	Receiver operating characteristics
RRT	Renal replacement therapy
sCr	Serum creatinine
SOPs	Standard operating procedures
TIMP-2	Tissue inhibitor metalloproteinase-2
